# Reducing access complications in an interdisciplinary structural heart program

**DOI:** 10.1186/s13019-025-03407-9

**Published:** 2025-04-02

**Authors:** Dinah Maria Berres, Markus Schlömicher, Boris Dickmann, Thomas Buck, Justus Thomas Strauch, Farhan Ahmad, Horatiu Coman, Peter Lukas Haldenwang

**Affiliations:** 1 Department of Cardiothoracic Surgery, BG University Hospital Bergmannsheil Ruhr-University of Bochum, Bürkle de la Camp-Platz 1, 44789 Bochum, Germany; 2 Department of Cardiology, Marien Hospital Witten, Witten, Germany; 3 Departement of Cardiology, Klinikum Westfalen Knappschaftskrankenhaus Dortmund, Dortmund, Germany; 4 Department of Cardiology, University Hospital Bergmannsheil, Bochum, Germany; 5 Department of Vascular Surgery, St. Josef-Hospital Bochum, Bochum, Germany; 6 Vascular Surgery Clinic, Cluj-Napoca County Emergency Hospital, Cluj-Napoca, Romania

**Keywords:** Aortic valve, Heart valve, transapical, percutaneous (TAVI), CardiaC, Outcomes (includes mortality, morbidity), Peripheral vascular disease, Artery/arteries

## Abstract

**Background:**

Vascular (VC) and cardiac structural complications (CSC) are frequent complications following transcatheter aortic valve implantation (TAVI). Aim of this single-center retrospective study was to evaluate strategies for minimizing periprocedural access complications as part of an interdisciplinary structural heart program.

**Methods:**

Included were all patients who underwent TAVI between 09/2022 and 08/2023 at our institution. All procedures were performed by a heart team, consisting of a cardiovascular surgeon with peripheral vascular and interventional experience and an interventional cardiologist on site. Valvular type and size, access route and backup strategies were assessed by the heart team according to the preoperative CT-imaging. Baseline characteristics, periprocedural data, complications and 30-day outcomes were analyzed concerning the access route using Mann-Whitney-U-test or Fisher´s exact test.

**Results:**

Analyzed were 167 consecutive patients (81 (76–85) years; 53.3% male). 48 (28.7%) of these had severe peripheral artery disease. 130 (77.8%) procedures were performed via a percutaneous transfemoral approach, 13 (7.8%) via a femoral cut-down and 4 (2.4%) via a transaxillary access. For 20 procedures (11.9%) a transapical access was used. 106 patients (72%) with transvascular and all patients with transapical access received a balloon-expanding valve, whereas 41 (28%) patients with transvascular access received a self-expanding prosthesis. No coronary occlusion was seen. Annular rupture occurred in one patient (0.6%), valve displacement in two patients (1.2%). Totally 5 (3%) access femoral arteries were stented and 8 (4.8%) needed a surgical reconstruction. 30-day mortality was 2.99%.

**Conclusions:**

On site interventional and cardiovascular surgical expertise may minimize VC and CSC following TAVI.

## Background

Over the last two decades transcatheter aortic valve implantation (TAVI) has become the therapy of choice for most patients with symptomatic aortic valve stenosis (AS) in the western hemisphere. In Germany, over 17,818 TAVI vs. only 7,798 surgical aortic valve replacements (SAVR) were performed in 2022 [1]. Regarding the chosen access way, transfemoral prior to transaxillary, transcarotideal or transaortic access is preferred, whereas a transapical approach via a left sided lateral mini-thoracotomy represents the last transcatheter option [2–6].

Vascular complications (VC) and cardiac structural complications (CSC) represent a serious safety limitation of these procedures, especially in elderly patients with chronic vascular disease. Although diagnostic tools, ultrasound-guided puncture techniques, percutaneous closure systems improved and vascular sheath diameter decreased over the last decade, VC and hemorrhage still occur in 6–8% following TAVI, being associated with an increased mortality, hospitalization and reduced quality of life [3, 7]. These complications may be best prevented and managed by a heart team with equivalent interventional and cardiovascular surgical expertise on-site in the hybrid operation room. As guidelines require only institutional on-site cardiac surgery, currently the interventional cardiologists perform most of the TAVI procedures without the physical presence of a cardiovascular surgeon. Aim of this single-center retrospective study was the evaluation of the prevention, early identification and effective management of VC and CSC in a heart team with equivalent interventional and vascular surgical expertise in the hybrid operating room.

## Materials and methods

Between 09/2022 and 08/2023 a total of 239 consecutive patients with severe AS were evaluated for TAVI vs. SAVR by the institutional heart team, consisting of a cardiovascular surgeon with peripheral vascular surgical and interventional experience, an interventional cardiologist and a cardio-anesthesiologist. Therapeutic decisions were patient-centered, based on cardiac and non-cardiac diagnostics, individual risk calculation (Society of Thoracic Surgeons Score, EuroSCORE II) and the patient’s frailty.

72 patients (30.1%) were found eligible for SAVR. For the remaining 167 patients (69.9%) a transcatheter procedure was considered. 147 (61.5%) consecutive patients were treated via a transvascular approach (TV-TAVI), whereas 20 (8.4%) patients received a transapical access (TA-TAVI).

### Preoperative diagnostics and vascular assessment

In the TAVI cohort, the preoperative diagnostics were completed by a coronary angiography and a contrast-enhanced computed tomography angiography (CTA) of the entire aorta (slice < 1 mm), including the supraaortic arterial branches, both iliacal and femoral arteries. Aortic valvular morphology (number of cusps, calcification pattern, annular-ostial distance, annular mean diameter, perimeter and area, diameter of the sinus of Valsalva and the sinutubular junction) as well as aortic and peripheric arterial morphology and calcification degree were assessed using 3mensio (Pie Medical, Maastricht, The Netherlands), Artis Pheno Syngo software (Siemens Healthineers, Erlangen, Germany) or OsiriX MD (Pixmeo SARL, Geneva, Switzerland) software.

Access vessel evaluation was done considering the minimal luminal diameter and identifying heavy iliacal or femoral calcification or calcification at the puncture site, the position of the femoral bifurcation and any significant vascular pathology like aneurysm, thrombosis, preexistent dissection or previous vascular interventions (endostent) or operations (bypasses, prosthetic interponates etc.).

**Fig. 1:**
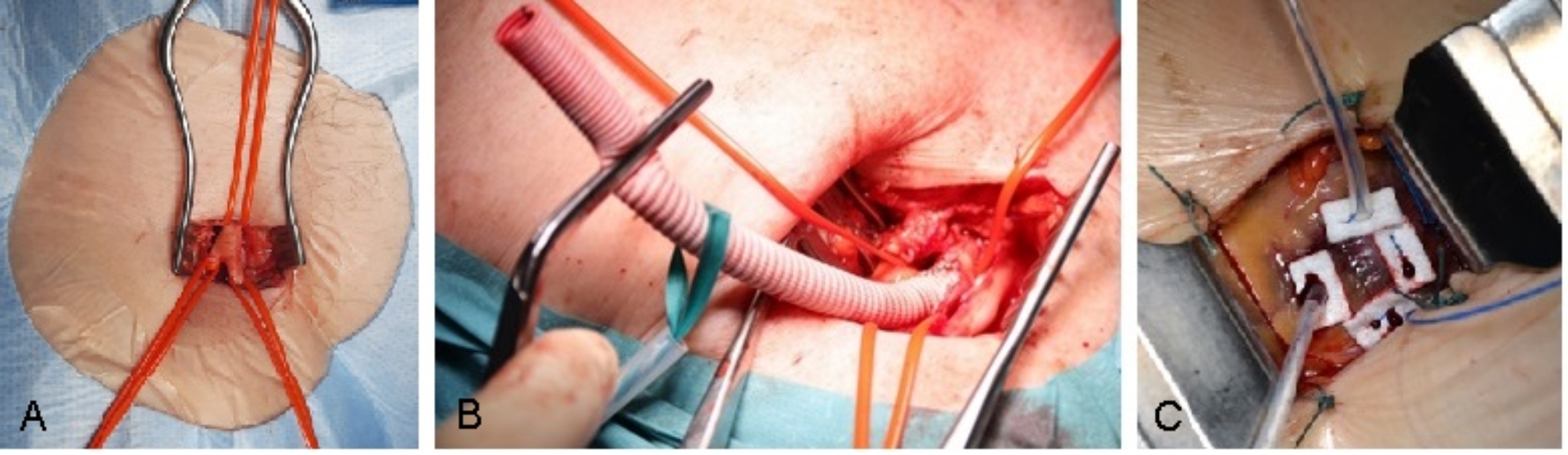
Open surgical access for TAVI. (**A**) Cut-down to the right femoral artery; (**B**) Exposure of the right axillary artery with end-to-side anastomosis of a vascular graft; (**C**) Exposure of the heart apex through a 5 cm mini-thoracotomy in the 4th or 5th intercostal space. Placement of two Teflon-enforced “U”-sutures in 90° for subsequent access way closure

**Fig. 2:**
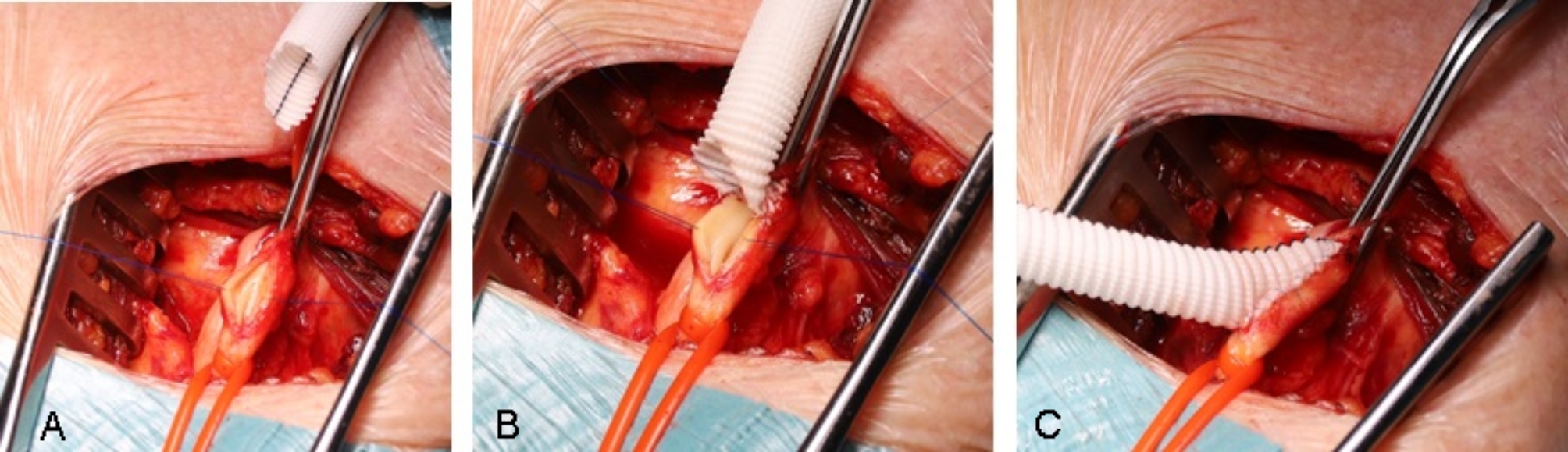
Transaxillary access via an end-to-side anastomosed conventional vascular graft Legend: (**A**) longitudinal incision of the left axillary artery; (**B**) and (**C**) End-to-side anastomosis of an 8 mm vascular graft for less traumatization of the naïve vessel during the TAVI procedure

The prosthetic valve type: balloon expanding (BE) SAPIEN-3 and SAPIEN-3-Ultra (Edwards Lifesciences Irvine, CA, USA) vs. self-expanding (SE) Medtronic Care Valve, Evolut R or Pro (Medtronic, Mineapolis, MN, USA) was decided based on the annular size, calcification pattern, age, preponderant pathology, vascular diameters and chosen access way. The optimal access way was determined for each patient according to the vascular situation in following order: transfemoral first, transaxillary second, transapical third. For transfemoral access, a transcutaneous ultrasound-guided puncture with application of suture-mediated closure systems was primary intended. If this this was considered not to be safe due to serious calcification at the puncture site, an open access via femoral cut-down was performed (Fig. 1A). For transaxillary and transapical approaches, open surgical exposures were considered as well (Fig. 1B and C) (Fig.  2).

### Implantation technique

Prosthetic valve implantation followed the standard protocol, respecting the special requirements for each valve type. If indicated, direct vascular access (cut-down) and/or vascular graft anastomosis as well as transapical access was provided by the cardiovascular surgeon. For transvascular TAVI, the position of the first implanter was alternatively assumed by the cardiovascular surgeon or the interventional cardiologist based on a 1:1 ratio. Transapical procedures were performed by the cardiovascular surgeon.

For a transfemoral approach, a percutaneous access with two Perclose™ Proglide™ (Abbott Medical, Wetzlar, Germany) suture-mediated vascular closure systems, placed at one and eleven o’clock was preferred. Alternatively, if a significant calcification at the puncture site led to a surgical cut down, a purse-string suture placed around the direct vascular puncture site was performed.

If transfemoral access was not feasible, because of small femoral or iliacal diameter, iliac stenosis and circular calcification or crossover femoral bypasses, the suitability for axillary access was assessed. For this approach, the left sided axillary artery was dissected and - in temporary clamping - an 8 mm conventional vascular graft (Gelweave, Vascutek/Terumo) was anastomosed with 6 − 0 Prolene running suture to the longitudinal incised native artery.

TAVI was then performed through this vascular prosthesis. Especially for patients treated with SAPIEN 3 valve prosthesis, a sufficient length of this vascular prosthesis enabled or simplified the later balloon pull-maneuver into the crimped valve, outside of the implantation sheath. For both, femoral and axillary access a minimum diameter of ≥ 5.5 mm was accepted. If this condition was not given, a transapical approach via a left sided lateral mini-thoracotomy at the apex level (double-checked via onsite fluoroscopy) was chosen. Before myocardial puncture, two Teflon felt reinforced U-sutures were placed transmural or deep into the myocardium, forming a “# “figure at the apex and the puncture and guiding wire placement set in its center. This was performed at a pharmacologically induced temporary systolic pressure drop to 80 mmHg to prevent excessive bleeding. As we need a vascular access for applicating the contrast agent, we also analyzed them with the vascular complications.

### Access management

**Fig. 3:**
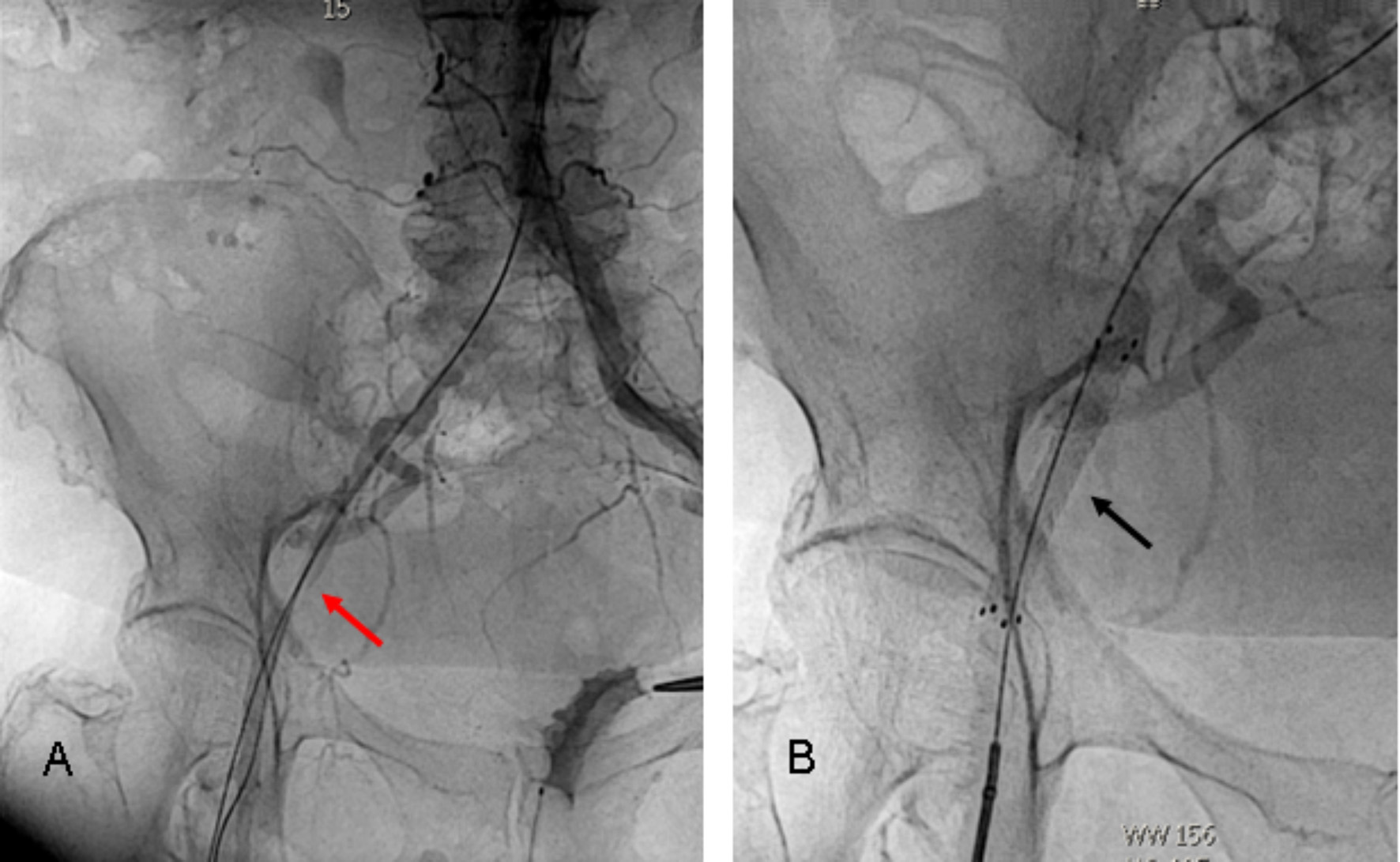
Femoral artery occlusion following suture-based percutaneous access way closure system (**A**) treated with an endostent (**B**) **A** – The vascular occlusion caused by the percutaneous closure system (red arrow), could be passed with a guidewire via a distal ipsilateral puncture. **B** – Femoral perfusion was restored via a self-expandable endostent (10 × 60 mm Fluency – black arrow)

**Fig. 4:**
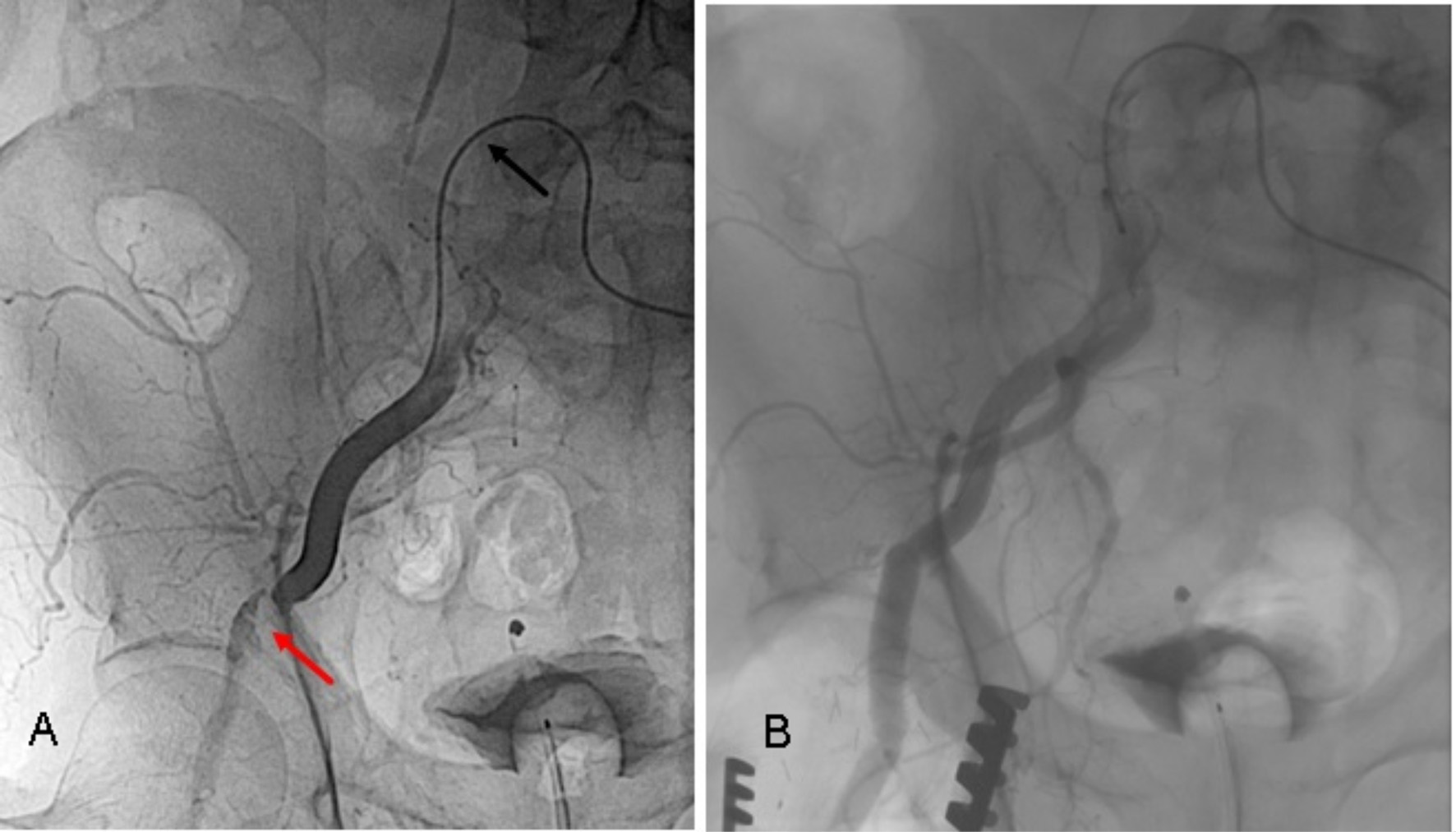
Post-TAVI femoral artery dissection/occlusion (**A**) treated with a vascular interponate (**B**) **A** – The vascular occlusion caused by the percutaneous closure system (red arrow), could not be passed in a crossover procedure (black arrow). **B** – Femoral perfusion was restored via an open surgical approach

After successful valve implantation, following closure scenarios were adopted: for a percutaneous transfemoral access, the 14–16 F implantation sheath was removed together with its inlay over the guiding wire to prevent a dissection or intimal injury. The puncture orifice was percutaneously closed by traction on both suture-mediated Perclose Proglide systems, without removing the guidewire. If this procedure already brought hemostasis, the guidewire was removed. If not an additional 6–8 F sheath was inserted over the guidewire to simulate a more expensive additional fibrin-mediated system (Angio-Seal 6–8 F, Terumo, Europe). If this could seal the leakage, the Angio-Seal was applied. If not, the inlay of the implantation sheath (14–16 F) was temporary reintroduced for hemostasis during decision-making. First choice was the placement of a self-expandable endovascular stentgraft (FLUENCY Plus, Bard Medical, USA), corresponding to the size of the common femoral artery via an ipsilateral distal femoral puncture (Fig. 3) or, more frequent, in crossover technique through the contralateral femoral artery. If this was not possible a surgical cut down was performed and the femoral or iliac vascular integrity restored by direct suture, a vessel patch or a vascular prosthetic interponate (Fig. 4). Finally, after the femoral arterial access way was closed, the iliac and femoral artery downstream was proven via digital subtraction angiography (DSA) and the contralateral access site closed percutaneously using a fibrin-mediated system (Angio-Seal 6 F, Terumo, Europe).

Following a subclavian or transaxillary TAVI, the implantation sheath was removed, the vascular graft double ligated at the level of its anastomosis to the native vessel and then shorted 0.5 cm above its ligature. The subclavian and axillary artery downstream was proven via DSA in the same manner as reported before.

For a transapical approach, the closure of the access site was performed by tightening the two apical purse-string sutures. If necessary, in case for a more fragile myocardial tissue, this was performed at a systolic pressure drop to 80 mmHg either induced pharmacologically or by temporary rapid pacing, in order to prevent bleeding complications.

### Vascular and access-related complications

VC and access-related were defined according to the latest Valve Academic Research Consortium-3 recommendations [4] (Table 1):

**Table 1:** Definition of vascular and Access-Related complications

Type of VC	Type of Injury	
Major VC	Aortic dissection or aortic rupture	
	Vascular injury or compartment syndrome	resulting in death, VARC type ≥ 2 bleeding, limb or visceral ischemia, or irreversible neurologic impairment
	Unplanned endovascular or surgical intervention	
	Closure device failure	
	Distal embolization (non-cerebral) from a vascular source resulting in death, amputation, limb or visceral ischemia, or irreversible end-organ damage	
Minor VC	Vascular injury or compartment syndrome	
	Unplanned endovascular or surgical intervention	
	Closure device failure	
	Distal embolization (non-cerebral) from a vascular source *not* resulting in death, amputation, limb or visceral ischemia, or irreversible end-organ damage	

### Cardiac structural complications

CSC were defined according to the latest Valve Academic Research Consortium-3 recommendations [4].

### In-hospital follow-up

Post-procedural neurological status was assessed by an independent neurologist, blinded to the procedure. Stroke was defined as the duration of a focal or global neurological deficit > 24 h or death due to neurological deficit. Both ischemic and hemorrhagic strokes were included and diagnosed with CT imaging.

### Primary and secondary endpoints

Primary endpoints were perioperative and 30-day VC and CSC following TAVI. Secondary endpoints were the 30-day mortality- and stroke-rate.

### Data presentation and statistics

The two-access way groups (TV-TAVI vs. TA-TAVI) were primary compared. Further, the outcome was compared regarding the type of the implanted prosthesis (BE vs. SE). Statistical analysis was performed using SPSS 18 software (SPSS Inc. Chicago, IL, USA). Continuous data with normally distribution were presented as mean ± standard deviation (SD), those with non-normally distribution as the median with the interquartile range (IQR), while categorical (qualitative) variables were summarized as counts (n) and percentages (%). Statistical significance was determined with Fisher’s exact test for categorical variables and the Mann-Whitney U-test for continuous variables. Significance was defined at a p-value < 0.05.

## Results

Regarding the preoperative data (Table 2) a significant difference was seen in age (median 82 (78–85) years for TV-TAVI vs. median 76 (72.75–80.25) years for TA-TAVI; *p* = 0.006) and preexisting severe peripheral vascular disease (19% for TV-TAVI vs. 100% for TA-TAVI; *p* < 0.005). From the total number of 147 transvascular TAVI patients, 106 (72%) received a BE and 41 (28%) a SE prosthesis. No differences were found for the two valve type subgroups regarding gender (54% male), age (median 82 years (78–85)) and STS Score (median 3.0% (1.8–4.6)). No other baseline data showed significant differences. 130 (77.8%) procedures were performed via a percutaneous transfemoral approach, 13 (7.8%) via a femoral cut-down and 4 (2.4%) via a transaxillary access using a vascular prosthetic graft-interponate. In five cases (3%) a safety wire was used (Table 3).

**Table 2:** Preoperative and demographic data

	*n*	transvascular	transapical	*p*-value
Patients	167	147 (88.1%)	20 (11.9%)	-
Male	89 (53.3%)	79 (53.7%)	10 (50%)	0.814
Age (y)	81 (76–85)	82 (78–85)	76 (72.75–80.25)	0.006*
BMI (kg/m ^2^)	26.7 (24.9–30.3)	27.7 (24.8–30.5)	25.3 (22.9–26)	0.200
Euroscore II (%)	3.6 (2.3-6)	3.4 (2.1–4.7)	4.2 (2.8–7.3)	0.047
STS Score (%)	3.0 (1.8–4.6)	2.8 (1.9–4.9)	3.1 (1.5–3.9)	0.65
CHD	116 (69.5%)	105 (71.4%)	11 (55%)	0.194
Pre PCI	79 (47.3%)	68 (46.3%)	11 (55%)	0.484
PVD	48 (28.7%)	28 (19%)	20 (100%)	< 0.005*
Pre VS	18 (10.8%)	12 (8.2%)	6 (30%)	0.01*
Pre LVEF (%)	50 (45–55)	50 (45–55)	50 (45–55)	0.21
Pre AVA (cm^2^)	0.8 (0.6–0.9)	0.8 (0.6–0.87)	0.8 (0.7–0.87)	0.67
Pre maxPG (mmHg)	54 (39–68)	52 (37.8–66.3)	57 (45-73.5)	0.54
BE-valve	126 (75.4%)	106 (72.1%)	20 (100%)	-

Data presented as n (%) or median ($\:IQR$), Discrete variables were analyzed with Fisher’s exact test for independent groups. Continuous variables were analyzed with the Mann–Whitney test for independent groups

*p < 0.05, BMI = Body Mass Index, CHD = Coronary Heart Disease, PCI = percutane coronary intervention, PVD = peripheral vascular disease, VS = vascular surgery, LVEF = left ventricular ejection fraction, AVA = aortic valve area, maxPG = maximum pressure gradient, BE = balloon expanding

**Table 3:** Access routes and preventive measures

Access Route Management	*n*
TF-access	143 (85.6%)
♣ TF-access percutaneous	130 (77.8%)
♣ TF-access via femoral cutdown	13 (7.8%)
TA-access	20 (11.9%)
TAx-access (prosthesis)	4 (2.4%)
Safety wire (femoral)	5 (3.0%)

Data presented as n (%), Discrete variables were analyzed with Fisher’s exact test for independent groups

*p < 0.05, TF = transfemoral, TA = transapical, Tax = transaxillary

Totally 5 (3%) access femoral arteries were stented and 8 (4.8%) needed a surgical reconstruction. The 30-day mortality was 2.99% (Table 4).

**Table 4:** Perioperative complications, morbidity and mortality

	*n*	transvascular	transapical	*p*-value
Patients	167	147 (88%)	20 (12%)	-
Valve displacement	2 (1.2%)	2 (1.4%)	0	1.0
Annular rupture	1 (0.6%)	1 (0.7%)	0	1.0
Coronary occlusion	0	0	0	-
Pericardial effusion	5 (3.0%)	5 (3.4%)	0	1.0
Stroke	5 (3.0%)	3 (2.0%)	2 (10%)	0.11
♣ Minor Stroke	2 (1.2%)	1 (0.7%)	1 (5%)	0.23
♣ Major Stroke	3 (1.8%)	2 (1.4%)	1 (5%)	0.32
Bleeding	15 (9.0%)	12 (8.2%)	3 (15%)	0.39
♣ Minor Bleeding	6 (3.6%)	5 (3.4%)	1 (5%)	0.54
♣ Major Bleeding	6 (3.6%)	5 (3.4%)	1 (5%)	0.54
♣ Life Threatening Bleeding	3 (1.8%)	2 (1.4%)	1 (5%)	0.32
Vascular complications	16 (9.6%)	15 (10.2%)	1 (5%)	0.67
♣ Minor VC	8 (4.8%)	8 (5.4%)	0	0.60
♣ Major VC	8 (4.8%)	7 (4.8%)	1 (5%)	1.0
♣ Vascular Stent	5 (3.0%)	5 (3.4%)	0	1.0
♣ Vascular Surgery	8 (4.8%)	7 (4.2%)	1 (5%)	1.0
30-day Mortality	5 (3.0%)	3 (2.0%)	2 (10%)	0.12
Intraoperative Mortality	1 (0.6%)	1 (0.7%)	0	1.0

Data presented as n (%), Discrete variables were analyzed with Fisher’s exact test for independent groups. *p < 0.05

Looking at the major vascular complications, no differences regarding valve size and consecutive arterial sheath size were seen. The only significant difference was detected in the “cutdown-access”-group – the group with the highest preexisting peripheral arterial comorbidity. One major vascular complication was seen in the TA-TAVI group. Nevertheless, the vascular complications had no significant influence on the length of ICU stay (0 vs. 1 day, *p* = 0.13) or the hospital stay overall (11 vs. 11.5 days *p* = 0.26) (Table 5).

**Table 5:** Type of vascular complications

	all	Non and minor	Major	*p*-value
Patients	167	159 (95.2%)	8 (4.8%)	-
ICU stay (d)	0 (0–1)	0 (0–1)	1 (0-3.25)	0.13
Hospital stay (d)	11 (9–13)	11 (9–13)	11.5 (11-15.5)	0.26
Valve size (mm)	26 (23–29)	23 (23–29)	23 (23–26)	0.07
Transfemoral access	143 (85.6%)	136 (85.5%)	7 (87.5%)	0.59
Transaxillary access	4 (2.4%)	4 (2.5%)	0	1.0
Transapical access	20 (12.0%)	19 (11.9%)	1 (12.5%)	0.12
Safety Wire	5 (3.0%)	5 (3.1%)	0	1.0
Cutdown Access femoral	13 (7.8%)	11 (6.9%)	2 (25%)	0.005*

Data presented as n (%) or median ($\:IQR$), Discrete variables were analyzed with Fisher’s exact test for independent groups. Continuous variables were analyzed with the Mann–Whitney test for independent groups. *p < 0.05

Per total two valve displacements occurred in all 167 transcatheter TAVI (1.2%). One could be solved interventionally by retracting the displaced valve into the ascending aorta and placing a second TAVI prosthesis in loco tipico. The second led to a surgical conversion. Both patients survived and had a good postoperative course. Further, one annular rupture occurred (0.6%). The procedure was converted immediately with emergent connection to extracorporeal circulation and full sternotomy, but the patient didn’t survive. No coronary occlusion - neither in the TV- nor in the TA-TAVI group was seen. Pericardial effusion happened in five patients (3.0%). Overall, five strokes (3.0%) occurred, with no significant differences between the two groups. Bleeding for any reason occurred in 15 patients (9%) of which six were only minor bleedings (3.6%).

## Discussion

In literature, the range of reported VC following TAVI is wide, reaching from 2.2% in the low-risk PARTNER-3 trial and 7.9% in the intermediate-risk PARTNER-2 trial to 27% in earlier high-risk TAVI trials [3, 7, 8]. A recently published multicenter, retrospective long-term study on more than 2000 patients who underwent TAVI, reported an VC incidence of 8.8%, from which 96% were limited to the access site, 2/3 were minor complications and 8% required a surgical treatment [9]. In a two-year perspective, MACCE-free survival was 71.9% for patients with major VC compared to 89% for those with minor VC. The authors conclude that major but not minor VC impact the long-term survival following TAVI [9]. A multicenter study with more than 3000 patients after TAVI saw major bleeding complications an independent increase in mortality as well as a decrease in quality of life after one-year follow-up [10]. This also shows the importance of an ongoing procedural improvement.

The major reasons for the widespread range of VC data are the inhomogeneous definitions for minor or major VC on one side and the discrepancy of the preoperative vascular morbidity in the analyzed cohorts on the other side. Another reason may be the evolution of transvascular techniques, ultrasound guided punctures, percutaneous closure systems and the decrease of sheath size and percutaneous valve delivery systems. In our cohort, the rate of major (4.8%) and minor (4.8%) vascular complications was low. Three of the VC could be treated conservatively with manual compression, five (3.4%) had a vascular stenting and eight (4.8%) needed open vascular surgery. In contrast, a primary femoral cut-down (6%) or a transaxillary access (3%) were used liberally. Nevertheless, these open surgical access ways presented no VC or VARC-3 type ≥ 2 bleedings at all, since they were performed under controlled, stable conditions. Nevertheless, neither minor VC nor major VC did affect the immobilization and hospitalization time.

In earlier studies circumferential vascular calcification at the access site, preexisting peripheral vascular disease, and female sex were identified as patient-related risk factors, whereas larger sheath sizes, increased sheath / femoral artery ratio, and implanters inexperience were considered non-patient-related risk factors for VC and bleedings [11–13]. Regarding the patient related risk factors such as age, female gender or preexisting PVD there were no significant differences between the groups. We also did not see any significant differences regarding ICU stay or hospital stay even if major vascular complications happened. The VARC-3 type > 2 bleeding rate of our cohort was with 5.4% lower than that reported by others [11].

As recently reported, CSC following TAVI decreased over the last years [11, 14]. In our series of 167 patients, the two valve displacements (1.2%) could be solved by the heart team: one interventionally and one surgically – both with a good result. Unfortunately, the annular rupture (0.6%) ended lethally, although a surgical correction with patch plastic and SAVR was performed immediately. Nevertheless, no coronary occlusion occurred. The assumption is, that a TAVI program with onsite interventional and surgical expertise enables prevention and a fast management of access site related complications.

### Limitations

This is a retrospective single-center study with a relatively small sample size. Also, it only reflects a single year experience. Unfortunately, we only had a small number of patients with transapical access. Although our intention was not to compare the transvascular with the transapical group but only demonstrate our way of decision making a greater sample size would have been more meaningful.

## Conclusion

VC and CSC prolong immobilization and hospitalization following TAVI. They represent a serious drawback, especially in elderly patients. To minimize these complications a rigorous preoperative access way planning as well as a fast intraoperative complication management are necessary. A primary interdisciplinary approach enables implanters to get routine in discovering patient related risk from different points of view.

## Data Availability

No datasets were generated or analysed during the current study.

## Abbreviations

AS: Aortic valve Stenosis
CSC: Cardiac Structural Complications
SAVR: Surgical Aortic Valve Repair
TA: Transapical
TAVI: Transcatheter Aortic Valve Implantation
Tax: Transaxillary
TV: Transvascular
VARC: Valve Academic Research Consortium
VC: Vascular Complications
